# Unexpected death after occipital condylar fracture

**DOI:** 10.1007/s00701-017-3165-5

**Published:** 2017-04-21

**Authors:** Vincent J. Bulthuis, E. M. J. Cornips, J. Dings, H. van Santbrink, A. A. Postma

**Affiliations:** 1 Department of Neurosurgery, Maastricht University Medical Center, Oxfordlaan 10, 6229 EV Maastricht, The Netherlands; 2 Department of Radiology, Maastricht University Medical Center, Maastricht, The Netherlands

**Keywords:** Clinical presentation, Complications, Epidural hematoma, Myelopathy, Occipital condylar fracture

## Abstract

We present a rare fatal complication of an occipital condylar fracture. The patient was initially neurologically intact, but showed secondary clinical deterioration. Imaging revealed extensive extra-axial hemorrhage at the craniocervical junction and an acute obstructive hydrocephalus. MR imaging demonstrated a T2 hyperintens signal in both the lower brainstem and upper cervical spinal cord, likely caused by the extra-axial hemorrhage. As prognosis was estimated infaust, supportive treatment was discontinued and the patient died soon thereafter. This case report illustrates a rare, delayed complication and unexpected death in a patient having sustained an occipital condylar fracture.

## Introduction

Occipital condylar fractures (OCFs) were considered quite rare, but widespread availability of modern neuro-imaging techniques has been revealing more cases in recent years. In most but not all cases, they are associated with severe craniocervical trauma such as a high speed motor vehicle accident or a fall from a substantial height [2]. The clinical presentation of OCFs is highly variable, though severe neurological deficits are more likely due to the associated neurotrauma [7]. We present a patient with an OCF and delayed neurological deterioration due to an extra-axial hemorrhage at the craniocervical junction with fatal injury to the brainstem and upper cervical spinal cord.

## Case description

**Fig. 1:**
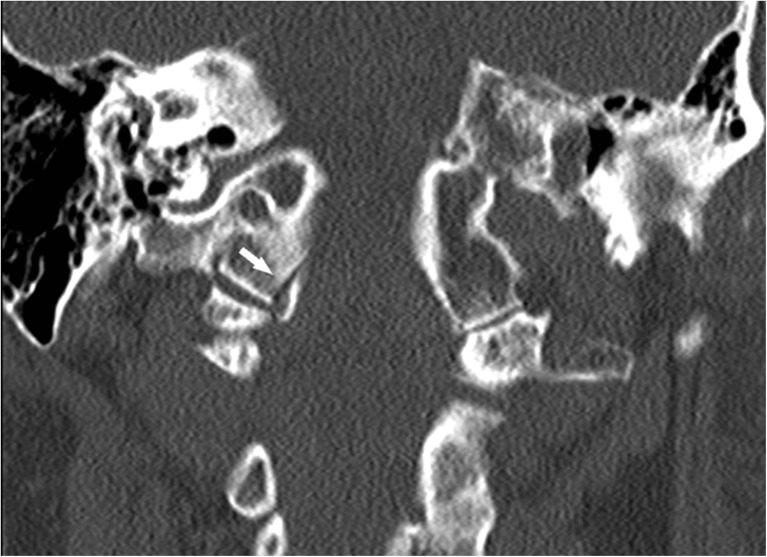
Coronal CT reconstruction of the craniocervical junction demonstrating an avulsion fracture of the right occipital condyl with inferomedial displacement of the fractured fragment (*white arrow*). The fracture is classified as an OCF type III according to Anderson and Montensano [1]

A 72-year-old female was admitted to the hospital after a fall down the stairs. Her medical history included diabetes mellitus, rheumatoid arthritis, and a prosthetic aortic valve. Her medication included warfarin and prednisolone. Her international normalized ratio (INR) on admittance (2.6) was corrected (1.1) with prothrombin complex concentrate and vitamin K. Her Glasgow Coma Scale score (GCS) on admission was maximal (15/15). Her main complaint was pain in the cervical and thoracic region. We did not observe any neurological symptoms or signs. We were unable to assess brain stem reflexes because of a massively swollen face; however, no swallowing disturbances and hoarseness, for example, were reported. CT scan demonstrated an Anderson and Montesano type III OCF [2] (Fig. 1), a C6 pedicle fracture, and a C7 facet fracture on the right side, as well as fractures of the T4 and T11 vertebral bodies, the T9 and T10 transverse process on the right side, and the T8–T11 transverse processes on the left side. We did not observe any fracture near the jugular foramen or hypoglossal canal or any intracranial abnormalities.

**Fig. 2:**
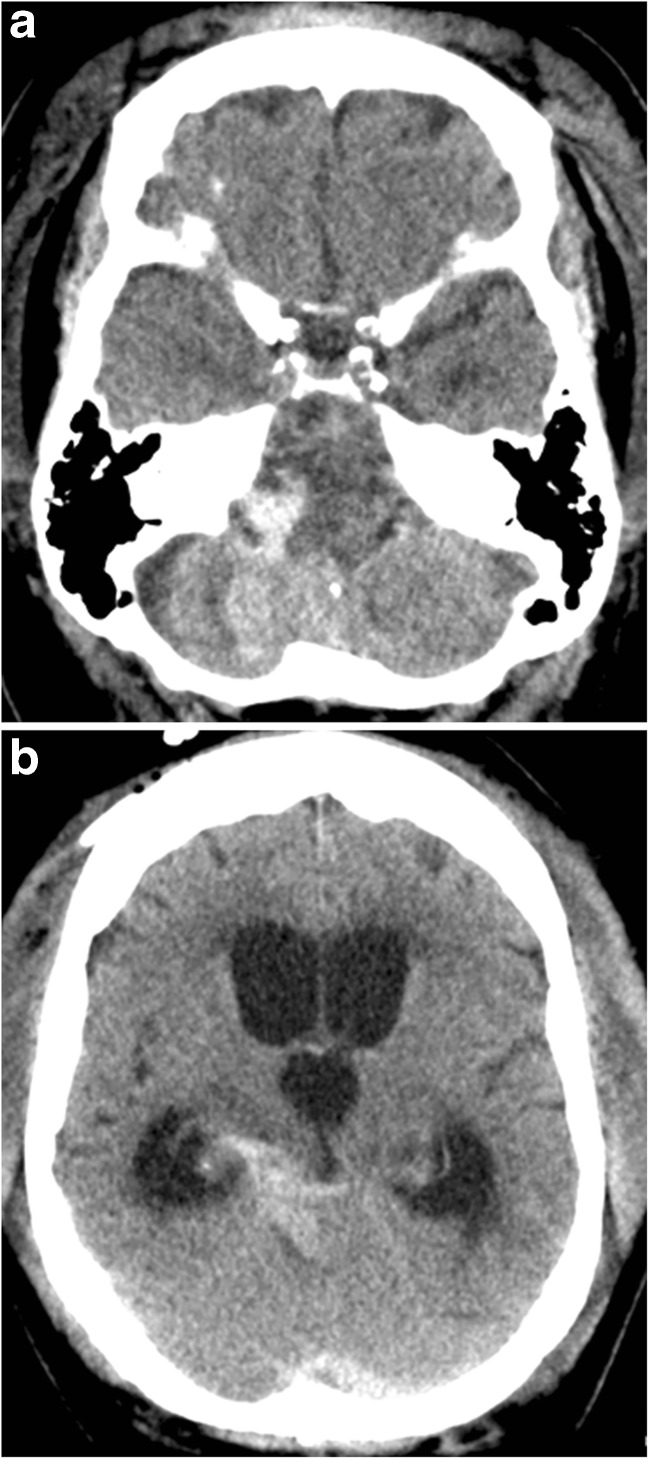
**a** and **b** Axial CT images of the brain demonstrate an extra-axial hemorrhage around the brainstem and in the posterior fossa, causing an acute obstructive hydrocephalus

**Fig. 3:**
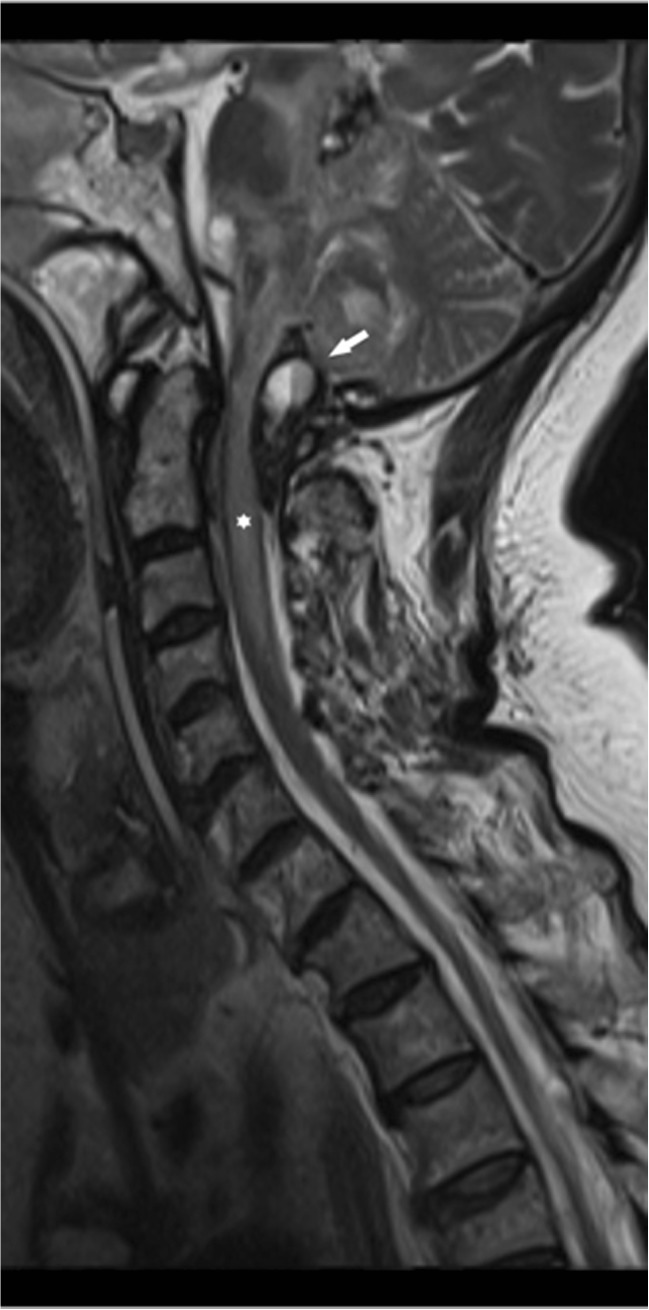
Sagittal T2-weighted MR image of the cervical spine. An epidural mass with fluid level, consistent with an acute hemorrhage, is compressing the lower brain stem and upper cervical spinal cord at the level of the foramen magnum (*white arrow*). Additionally, hemorrhage is present at the level of the supracerebellar cistern. Note an increased signal intensity in the entire brain stem, upper cervical spinal cord (to the level of C4) (*white asterisk*), and cerebellum

HALO vest immobilization seemed appropriate for the OCF, but was practically impossible because of the massively swollen face. Hence, the patient was admitted to the neurosurgical ward with a rigid cervical collar. Two days after admission, she was found comatose in bed (Glasgow Coma Scale score 7/15). While we were still unable to assess brain stem reflexes, we did observe an extensor plantar response (Babinski sign) and triple response bilaterally. Emergency CT scan demonstrated an extra-axial hemorrhage around the brainstem and in the posterior fossa causing an acute obstructive hydrocephalus (Fig. 2a, b). Emergency external ventricular drainage did not improve the patient’s clinical condition, as her Glasgow Coma Scale score was 3/15 without any reaction to peripheral stimuli, while being unsedated and ventilated without any reaction. Next morning, MR imaging demonstrated a T2 hyperintens signal in both the lower brainstem and upper cervical spinal cord (C0 to C5) likely caused by the extra-axial hemorrhage compressing the brainstem and obstructing the cerebrospinal fluid flow over the foramen magnum (Fig. 3). The observed anomalies clearly explained the patient’s clinical condition, including tetraplegia and acute respiratory failure. Due to the extent of neurological damage and the duration of symptoms, prognosis was estimated infaust, and any surgical intervention was deemed futile. Supportive therapy was therefore discontinued, and the patient died soon thereafter.

## Discussion

First described in 1817 by Bell [3], OCFs were considered quite rare. While their exact incidence is still unknown, they are being recognized more often because of an increased use of CT and MR imaging [16]. To date, their incidence in severely injured trauma patients is estimated between 1% and 2% [7, 14]. The most widely used classification is the one by Anderson and Montesano [2] who divided OCFs into three subtypes. A type I is an impaction fracture of the occipital condyle caused by an axial loading injury. A type II is part of a more extensive skull base fracture (involving one or both occipital condyles) often caused by a direct blow to the skull. Both types are considered stable fractures [1] because of the intact alar ligaments and tectorial membrane. A type III is an avulsion fracture of the occipital condyle caused by stress on the ipsi- and contralateral alar ligaments and tectorial membrane following forceful hyperextension or hyperflexion at the craniocervical junction. It is considered a potentially unstable fracture [7].

Due to a small number of patients and a lack of prospective studies, the management of OCFs is not well established [8, 16]. Most patients with an Anderson and Montesano type I or II OCF are treated with a rigid collar, while most patients with a type III fracture are treated with a rigid collar, a HALO vest, or even surgical fixation [4, 7].

Even though there is substantial variability in the clinical presentation of OCFs, they always point to a serious impact to the posterior skull base and craniocervical junction. They have been associated with cranial nerve palsies (in casu the lower cranial nerves), tetraplegia, and even fatal brainstem contusion. Cranial nerve palsies are among the most frequently described injuries with an incidence as high as 31% according to some authors [15]. Nerve injury may range from an isolated cranial nerve palsy [11, 13] to uni- or bilateral complete 9th through 12th cranial nerve palsies (the so-called Collet-Sicard syndrome) [5].

Despite the substantial impact necessary to fracture an occipital condyle, associated extra- or subdural hemorrhage at the craniocervical junction seems to be quite rare. In an analysis of 100 patients with 106 OCFs, merely 5 patients manifested an extra-axial hematoma on MR imaging [9]. On the other hand, in a recent review by Theodore et al., as many as 13 (22%) of 59 patients with an OCF manifested an extra-axial hemorrhage at the craniocervical junction [14]. This discrepancy in the reported incidence of this hemorrhage may be explained by the fact that the hemorrhage may be asymptomatic. Of note, both papers do not specify the clinical course, eventual complications, and final outcome of these patients [9, 14]. Finally, three case reports illustrate the occurrence of an epidural hematoma anterior to the upper cervical spinal cord in association with an OCF [4, 6, 12]. These patients invariably suffered lower cranial nerve palsies.

Both the occurrence of hemorrhage at the craniocervical junction and lower cranial nerve palsies may be explained by the close relationship of the occipital condyle to the hypoglossal canal and jugular foramen, both of which contain an artery, a vein, and one or several cranial nerves [6]. More specifically, through the hypoglossal canal at the occipital condylar base pass the hypoglossal nerve (XIIth nerve), a meningeal branch of the ascending pharyngeal artery, and an emissary vein, while through the jugular foramen (lateral to the occipital condyle and hypoglossal canal) pass cranial nerves IX, X, and XI, the posterior meningeal artery, the inferior petrosal sinus, and the sigmoid sinus on its way to the internal jugular vein bulb [6, 8, 10]. Vascular and cranial nerve injuries in patients with an OCF are likely caused by displacement of the fractured condylar fragment in close vicinity to these fragile neurovascular structures [5]. Alternatively, venous oozing from a fractured skull base may cause increased local pressure by accumulation of fluids from the neighboring vessels due to an osmotic pressure gradient across the hematoma capsule (hygroscopic effect). The use of both warfarin and prednisolone may have contributed to the posttraumatic hemorrhage in this patient.

The secondary clinical deterioration and subsequent death of this patient may be explained by a developing myelopathy following direct compression on the lower brainstem and upper cervical spinal cord by an extradural hematoma. This irreversible damage shows the vulnerability of the craniocervical junction.

In retrospect, we could have considered performing an additional MR cerebrum and craniocervical junction following the patient’s clinical deterioration to evaluate injury of the ligaments and possible local compression of the brainstem and upper cervical spinal cord. Such additional imaging might have contributed to an earlier understanding of what was actually causing the acute hydrocephalus. Of note, an early CT angiography or CT cerebrum may have given valuable information in addition to the clinical picture as well; however, both would not have been useful for a detailed assessment of the craniocervical junction.

## Conclusion

An occipital condylar fracture is a potentially life-threatening condition because of the associated neurovascular damage. Symptoms may be present in the acute phase or develop in the first few days, causing dramatic clinical deterioration.

CT, computed tomography; MR, magnetic resonance; OCF, occipital condylar fracture.
